# MACC1 regulates PDL1 expression and tumor immunity through the c‐Met/AKT/mTOR pathway in gastric cancer cells

**DOI:** 10.1002/cam4.2542

**Published:** 2019-09-26

**Authors:** Gangling Tong, Boran Cheng, Jingzhang Li, Xuan Wu, Qiaohong Nong, Lirui He, Xi Li, Laiqing Li, Shubin Wang

**Affiliations:** ^1^ Department of Oncology Peking University Shenzhen Hospital Shenzhen China; ^2^ Department of Oncology Liuzhou People's Hospital Liuzhou China; ^3^ Guangzhou Youdi Bio‐technology Co., Ltd Guangzhou China

**Keywords:** c‐Met/AKT/mTOR pathway, gastric cancer, MACC1, PDL1, tumor immunity

## Abstract

**Background:**

Immunotherapy and its mechanisms are being studied in a wide variety of cancers. Programmed cell death ligand 1 (PDL1) is associated with immune evasion in numerous tumor types. Here, we aimed to assess the relationship between metastasis associated in colon cancer‐1 (MACC1) and PDL1 and examine their effects on gastric cancer (GC) tumor immunity.

**Methods:**

The expression of MACC1, c‐Met, and PDL1 in human GC tissues was first assessed using quantitative RT‐PCR (qRT‐PCR) and immunohistochemistry. We then focused on the relationships among MACC1, c‐Met, and PDL1 using RT‐PCR and western blotting after cell transfection and inhibitor treatment in vitro and on the identification of their roles in immune killing in vitro and in vivo.

**Results:**

We found that expression of MACC1, c‐Met, and PDL1 was upregulated in human GC tissues, and there was a positive correlation between the expression levels. In addition, we found that ectopic expression of MACC1 (silencing and overexpression by transfection) resulted in corresponding changes in c‐Met and PDL1 expression levels, and c‐Met/AKT/mTOR pathway inhibitors (SU11274, MK2206, and rapamycin) blocked the regulation of PDL1 expression by MACC1. Furthermore, silencing of MACC1 led to an increase in antitumor and immune killing in vitro and in vivo, and overexpression of MACC1 resulted in a decrease in tumor immunity in vitro and in vivo.

**Conclusions:**

From these data, we infer that MACC1 regulates PDL1 expression and tumor immunity through the c‐Met/AKT/mTOR pathway in GC cells and suggest that MACC1 may be a therapeutic target for GC immunotherapy.

## Graphical abstract

MACC1 regulates PDL1 expression and gastric cancer tumor immunity. MACC1 regulates gastric cancer tumor immunity via the c‐Met/AKT/mTOR pathway. MACC1 may be a therapeutic target for gastric cancer immunotherapy.

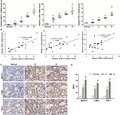

## 1 INTRODUCTION

Numerous clinical surveys and statistics have found that gastric cancer (GC) is one of the most common malignant gastrointestinal tumors and has a significant impact on global cancer death.^1^ According to statistics, for patients with advanced GC who have undergone advanced systemic therapy, the 5‐year overall survival rate does not exceed 20%.^2^ The etiology of GC is a complex process involving multiple genes, and mutation and loss of tumor suppressor genes can promote tumor progression.^3^ Therefore, it is important to study the mechanism of GC progression and alternative therapies. In addition to surgical treatment, radiotherapy, and chemotherapy, gene therapy and immunotherapy are being studied.^4^, ^5^

In the regulation of immune responses, many studies have inferred that the programmed cell death‐1 (PD‐1)/programmed cell death ligand 1 (PDL1) pathway has important roles. In treating many types of cancer, immunotherapy targeting PD‐1/PDL1 is a breakthrough and has great promise in cancer treatment^5^ In 2015, The US FDA approved anti‐PD‐1 monoclonal antibodies nivolumab and pembrolizumab for advanced non‐small cell lung cancer treatment, which achieved a good therapeutic effect.^6^, ^7^ Both antigen‐presenting cells and cancer cells can express PDL1, and PDL1 expression of numerous tumor is associated with immune evasion.^8^ Zou et al noted that overexpression of PDL1 may be one mechanism by which tumor cells escape the PD‐1/PDL1 immune inhibitory pathway.^9^, ^10^ Therefore, it is necessary to study the regulatory gene of PDL1 and its regulatory pathway.

Our previous study found that the expression of MACC1 and PDL1 was upregulated in GC. From this discovery and previous research, we wondered whether there was a correlation between MACC1 and PDL1 and what role MACC1 plays in tumor immune evasion. Using MACC1 transfection and inhibitor treatment, this study aimed to verify the relationship between MACC1 and PDL1 and examine their effects on GC tumor immunity.

## 2 MATERIALS AND METHODS

### 2.1 Clinical samples and gastric cancer cell lines

Clinical tissue samples were obtained between 2015 and 2016 at Peking University Shenzhen Hospital (Shenzhen, China). All tissue samples were partly fixed in 4% paraformaldehyde solution for immunohistochemistry and partly freshly frozen in liquid nitrogen and stored at −80°C. The human GC cell lines, MKN45 and MKN28, were obtained from the Shanghai Cell Collection. Cells were cultured in a 5% CO_2_ humidified atmosphere at 37°C with Dulbecco's modified Eagle's medium (DMEM) supplemented with 10% fetal bovine serum (FBS).

### 2.2 Quantitative real‐time PCR (qRT‐PCR) assay

After total RNA extraction by TRIzol Reagent (Invitrogen), the cDNA was synthesized using A cDNA Synthesis Kit (Takara). qRT‐PCR detected the mRNA levels of MACC1 and PDL1 mRNA using the LightCycler 480 detection system (Roche Diagnostics, USA). The mRNA levels were normalized to the levels of *β*‐actin mRNA. The primer sequences used in this study are presented in Table 1. Relative mRNA levels based on the CT value were used for qRT‐PCR results analysis and expression.

**Table 1:** Primers used for quantitative real‐time PCR analysis. Primers were designed using Primer Express version 2.0 software (Thermo Fisher). Primer specificity was confirmed using Primer‐BLAST web software (National Centre for Biotechnology Information)

Genes	Names	Primers (5ʹ to 3 ʹ)
MACC1	MACC1‐F	GTCATGTGGCTGTGGGAGAA
	MACC1‐R	TTTCCAACAACGGGCTCACA
c‐Met	c‐Met‐F	TGGGCACCGAAAGATAAACCT
	c‐Met‐R	TCGGACTTTGCTAGTGCCTC
IFN‐*γ*	IFN‐*γ*‐F	CTGTTACTGCCAGGACCCAT
	IFN‐*γ*‐R	TCTGTCACTCTCCTCTTTCCA
IL‐10	IL‐10‐F	TTGCTGGAGGACTTTAAGGGTT
	IL‐10‐R	TCACATGCGCCTTGATGTCT
IL‐2	IL‐2‐F	CAAACCTCTGGAGGAAGTGCT
	IL‐2‐R	TGTTGTTTCAGATCCCTTTAGTTC
PDL1	PDL1‐F	TTGCTGAACGCCCCATACAA
	PDL1‐R	GTCCAGATGACTTCGGCCTT
GAPDH	GAPDH‐F	ACAACTTTGGTATCGTGGAAGG
	GAPDH‐R	GCCATCACGCCACAGTTTC

### 2.3 Immunohistochemical assay

Firstly, 3‐5 μm tissue sections were incubated at 56°C for 2 hours and then deparaffinized. Following hydration and blocking, the slides were treated with peroxide. The target primary antibodies were incubated overnight. After washing with PBS and incubating with secondary antibody for 30 minutes at 37°C, the slides were treated with 3,3ʹ‐diaminobenzidine (DAB) solution. Finally, the slides were counterstained lightly with hematoxylin and dehydrated. Olympus fluorescence microscope (Olympus, CX71) photographed at 400× magnification, and Image‐Pro Plus software (Media Cybernetics) analyzed the medium optical density (MOD).

### 2.4 Cell transfection

For the overexpression of MACC1 in MKN28 cells, the ectopic MACC1 coding sequence was amplified by PCR and cloned into the pCDNA3.1(+) vector. For MKN45 cells with MACC1 silencing, short hairpin RNA targeting MACC1 (shMACC1) sequences and its negative control (shMACC1‐NC) sequences were integrated into the pSUPER‐retro‐puromycin plasmid (Table 2). The above plasmids were combined with PIK vector or blank pCDNA3.1(+) vector, and the lentiviral vectors were constructed. Using Lipofectamine 2000 (Invitrogen, Carlsbad, CA), MKN28 and MKN45 cells were transfected with lentiviral vectors. The transfected cells were selected with G418 and puromycin for 48‐72 hours. After monoclonal screening, the stably transfected cell lines were prepared.

**Table 2:** Primers used for cells silencing and overexpression transfection. Primers were designed using Primer 5.0 software

Genes	Names	Primers (5 ʹ to 3 ʹ)
shMACC1	shMACC1‐sense	CACCATGGCTTGGTTAAGTCAACCGAAGTTGACTTAACCAAGCCA
shMACC1‐NC	shMACC1 control‐sense	CACCGTTCTCCGAACGTGTCACGTCGAAACGTGACACGTTCGGAGAA
Vector‐NC	Vector control	Blank pCDNA3.1(+) vector
MACC1	Code sequence	NM_182762

### 2.5 Western blot assay

After washed with PBS and lysed in radioimmunoprecipitation assay buffer, the concentrations of total protein were measured using BCA Protein Detection Kit (Youdi Biotech, Guangzhou, China). Then, 50‐µg total protein was resolved by 12% SDS‐PAGE and transferred to a polyvinylidene difluoride membrane (Millipore, Germany). Following blocking, immunoblot detection and visualization were performed with enhanced chemiluminescence reagents (Pierce; Thermo Fisher Scientific, Inc). Immunoblotting was performed with target primary antibodies. GAPDH levels were used for normalization. ChemiDoc image analysis system (Bio‐Rad Laboratories, Inc) analyzed and quantified the relative protein levels.

### 2.6 Inhibitor treatment

After trypsinization, 5 × 10^5^ transfected MKN28 cells were seeded on 6‐well plates for 12 hour. After 24 hours of serum deprivation, SU11274 (phosphorylated c‐Met (p‐c‐Met) inhibitor; Selleck Chemicals, Houston, TX, USA; 5 μmol/L), MK2206 (phosphorylated AKT (p‐AKT) inhibitor; Santa Cruz Biotechnology, Dallas, TX, USA; 5 μmol/L), and rapamycin (phosphorylated mTOR (p‐mTOR) inhibitor; Selleck Chemicals, Houston, TX, USA; 200 nmol/L) were added to the 6‐well plates and cocultured for 24 hours. Then, the cells were collected and probed by western blot assay.

### 2.7 Peripheral blood mononuclear cell (PBMC) isolation and culture

Fifteen milliliter whole blood was anticoagulated with ethylenediaminetetraacetic acid (EDTA), mixed with pH 7.4 PBS 1:1, layered over Ficoll Paque PLUS solution (GE Healthcare Life Sciences) (20 mL), and centrifuged at 400 × *g* for 40 minutes. PBMCs were isolated and cultured in DMEM supplemented with 10% FBS, 20 000 U/L IL‐2, 0.4 mg/L CD28, and 0.5 nmol/L CpG ODN (sequences: 5ʹ‐TCCATGACGTTCCTGACGTT‐3ʹ) in a 5% CO_2_ humidified atmosphere at 37°C. After 2‐3 days of activation, PBMCs were used for tumor cell immune killing analysis.

### 2.8 Cell viability assay (MTS)

After trypsinization, 4 × 10^4^ PBMC cells and 2 × 10^3^ transfected MKN45 (or MKN28) cells were cocultured on 96‐well plates. The cell viability was measured by 3‐(4,5‐carboxymethoxyphenyl)‐2‐(4‐sulfophenyl)‐2H‐tetrazolium (MTS) assay on days 0, 1, 2, 3, and 4. For this, 20 μL of CellTiter 96® AQueous One Solution Reagent (Promega) was added to each well and incubated for 2 hours in a 5% CO_2_ humidified atmosphere at 37°C. Thereafter, the optical density was measured by microplate reader (Thermo Scientific) at 490 nm. All experiments were performed in triplicate. Tumor cell killing rate = [1 − (OD_experiment_ − OD_PBMC_)/OD_tumor cells_] × 100%.

### 2.9 In vivo xenografting assay

To evaluate the roles of MACC1 in vivo, MACC1‐silenced MKN45 cells and their control cells or MACC1‐overexpressing MKN28 cells and their control cells (1 × 10^6^ cells in 200 μL serum‐free medium) were subcutaneously and bilaterally inoculated into the flank regions of legs of 6‐week‐old male nude mice, four mice each group. The length and width of tumor tissues were monitored every 3 days using caliper. Tumor volumes were calculated according to the formula: Volume = width × length × (width + length)/2. On day 28, after blood collection, the mice were euthanized, and tumor tissues were isolated and weighed. The target protein expression levels were detected using a western blot assay.

### 2.10 Quantitative detection of the cytokines by ELISA

The secretion of interleukin‐2 (IL‐2), interleukin‐10 (IL‐10), tumor necrosis factor‐*α* (TNF‐*α*), and interferon‐*γ* (IFN‐*γ*) were assessed by ELISA kits. The human IL‐2 ELISA Kit (Abcam, ab174444), human IL‐10 ELISA Kit (Abcam, ab46034), human TNF‐*α* ELISA Kit (Abcam, ab181421), and human IFN‐*γ* ELISA Kit (Abcam, ab46025) were used to detect the concentrations in the cell supernatant. The mouse IL‐2 ELISA Kit (Abcam, ab100716), mouse TNF‐*α* ELISA Kit (Abcam, ab100747), and mouse IFN‐*γ* ELISA Kit (Abcam, ab100689) were used to determine the concentrations in the mouse serum samples. The absorbance (OD) was measured at a wavelength of 450 nm.

### 2.11 Statistical methods

Statistical analysis was performed using SPSS 17.0 statistical software (IBM SPSS). Student's *t* test was used to determine statistically significant differences between groups. Multiple comparisons were made among ≥ 3 groups using one‐way ANOVA followed by the Bonferroni post hoc test. The nonparametric Mann‐Whitney U test was used if data were not normally distributed. The data are presented as the mean ± SD and *P* < .05 indicates a statistically significant difference.

## 3 RESULTS

### 3.1 MACC1 expression patterns in clinical gastric cancer tissue samples

**Figure 1:**
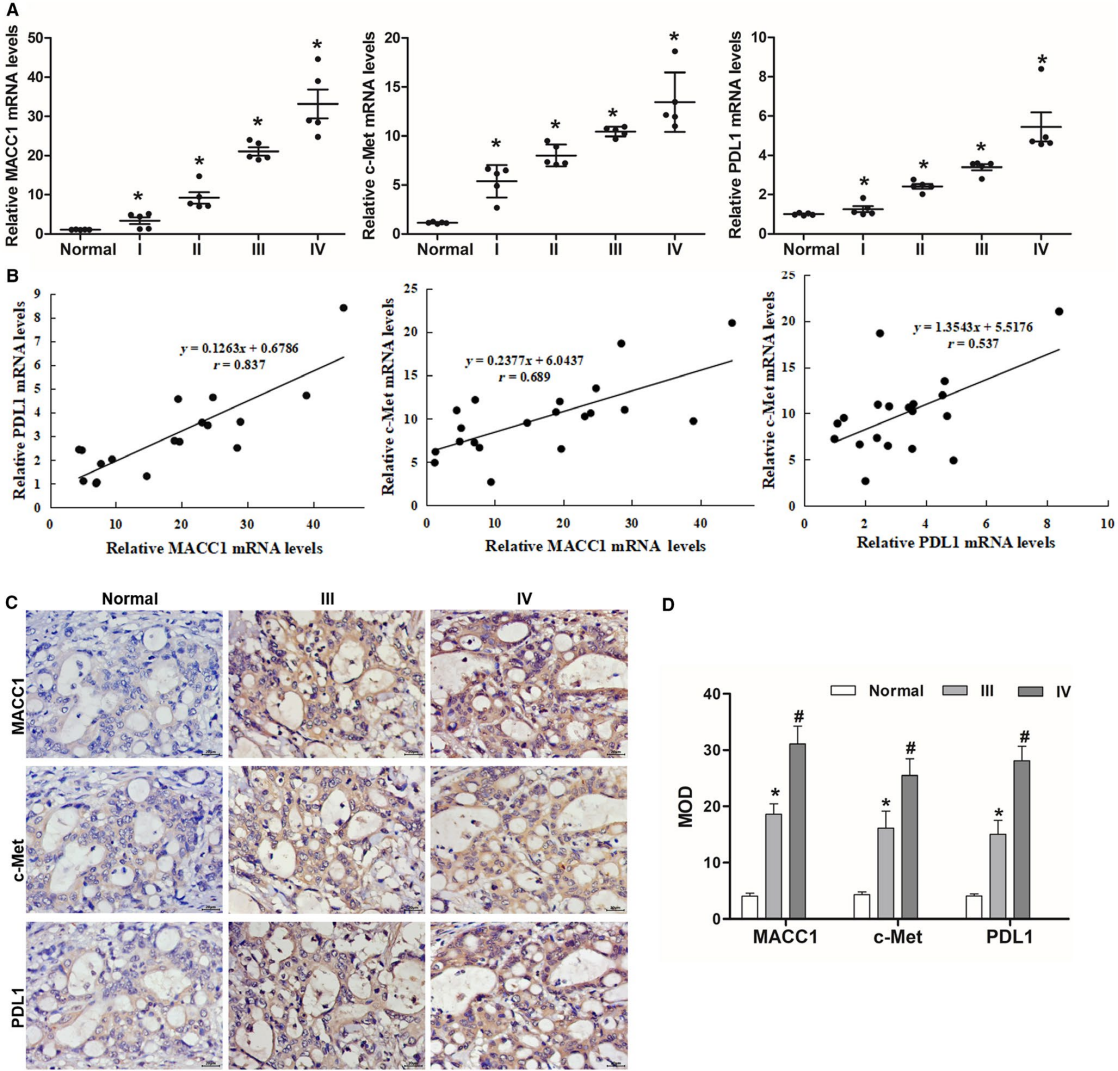
Expression of MACC1, c‐Met, and PDL1 in gastric cancer tissue samples and cell lines. (A) qRT‐PCR analysis of MACC1, c‐Met, and PDL1 mRNA expression. *P < .05, compared to abnormal tissues and cells. (B) Correlation analysis of MACC1, c‐Met, and PDL1 expression. (C) Immunohistochemical analysis of MACC1, c‐Met, and PDL1 proteins. (D) The medium optical density (MOD) statistical results of MACC1, c‐Met, and PDL1. *P < .05 and ^#^ *P* < .05, compared to abnormal tissues. Data are the mean ± SD (n = 3), and each bar represents the mean of three independent experiments carried out in triplicate

We simultaneously analyzed MACC1, c‐Met, and PDL1 mRNA expression patterns in whole‐genome gene profiles of 20 GC tissue samples, with the results shown in Figure 1A. MACC1, c‐Met, and PDL1 mRNA expressions were significantly higher in GC tumor tissues than in normal gastric mucosa tissues, and their mRNA expression levels were significantly associated with tumor grades (grades II, III, and IV, *P* < .05). Furthermore, Pearson's correlation assay indicated that there was a positive correlation between MACC1 and c‐Met expression levels, MACC1 and PDL1 expression levels, and c‐Met and PDL1 expression levels (Figure 1B). In addition, an immunohistochemical assay (Figure 1C,D) showed that MACC1 proteins were expressed in the nucleus and cytoplasm, c‐Met proteins were strongly expressed in the cytoplasm and extracellular matrix, and PDL1 proteins were markedly expressed in the membrane compared with the low expression of MACC1, c‐Met, and PDL1 in normal gastric mucosa tissues. The correlation analysis between expression levels of MACC1, c‑Met, and PDL1 and the clinicopathological parameters of gastric tumors found that the high MACC1, c‑Met, and PDL1 expression levels were associated with metastasis, tumor node metastasis stage, and life span (Table 3). We infer from these results that there may be some type of regulatory relationship among MACC1, c‐Met, and PDL1.

**Table 3:** Correlation analysis between expression levels of MACC1, c‑Met, and PDL1 and the clinicopathological parameters of gastric tumors

Clinicopathological characteristics	Patients, n	MACC1 expression		*P*	c‐Met expression		*P*	PDL1 expression		*P*
		−	+		−	+		−	+	
Sex				.15			.26			.54
Male	14	2	12		1	13		2	12	
Female	6	0	6		0	6		2	4	
Age, years				.32			.51			.76
< 60	12	2	10		0	12		4	8	
≥60	8	0	8		1	7		0	8	
Tumor size, cm				.056			.005			.14
<5	9	2	7		1	8		4	5	
≥5	11	0	11		0	11		0	11	
Lymph metastasis				.002			.001			.004
−	12	2	10		1	11		4	8	
+	8	0	8		0	8		0	8	
Peritoneal metastasis				<.001			.002			<.001
−	16	2	14		1	15		4	12	
+	4	0	4		0	4		0	4	
TNM stage				<.001			<.001			<.001
I	5	2	3		1	4		4	1	
II	5	0	5		0	5		0	5	
III	5	0	5		0	5		0	5	
IV	5	0	5		0	5		0	5	
Overall survival, years				.063			<.001			.003
<5	8	0	8		0	8		0	8	
≥5	12	2	10		1	11		4	8	
Disease‐free survival, years				.001			<.001			<.001
<5	9	0	9		0	9		0	9	
≥5	11	2	9		1	10		4	7	
Location				.058			.14			.21
Cardia‐fundus + gastric body	7	1	6		1	6		3	4	
Antrum	13	1	12		0	13		1	12	
Expression				<.001			<.001			<.001
Tumor tissues	20	2	18		1	19		4	16	
Adjacent normal tissues	13	0	13		0	13		0	13	

TNM, tumor node metastasis.

### 3.2 Ectopic expression of MACC1 regulates c‐Met and PDL1 expression in gastric cancer cells in vitro

**Figure 2:**
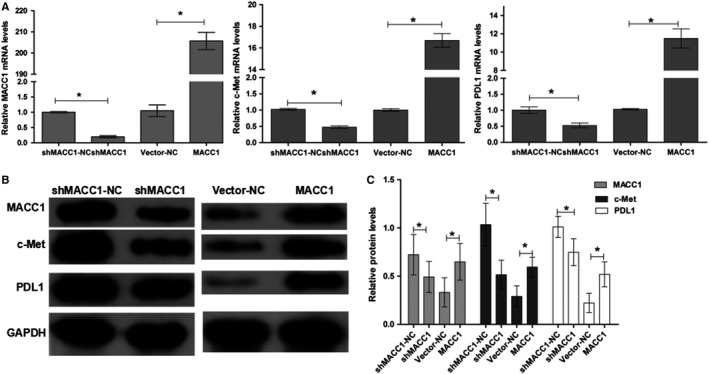
Expression of MACC1, c‐Met, and PDL1 in gastric cancer cell lines after MACC1 silencing or overexpression. (A) mRNA expression levels of MACC1, c‐Met, and PDL1 after transfection. *P < .05, compared to the shMACC1‐NC group. (B) Western blot analysis of MACC1, c‐Met, and PDL1 proteins expression. (C) Relative protein levels of MACC1, c‐Met, and PDL1. *P < .05, compared to shMACC1‐NC or vector‐NC. The relative expression of MACC1, c‐Met, and PDL1 is presented as the ratio of the expression level of GAPDH. Data are the mean ± SD (n = 3), and each bar represents the mean of three independent experiments performed in triplicate

To verify our hypothesis, we simultaneously designed and carried out silencing and overexpression of MACC1. After MACC1 knockdown transfection in MKN45 cells, RT‐PCR indicated that the expression levels of MACC1, c‐Met, and PDL1 were significantly downregulated compared with the shMACC1‐NC group. The expression levels of MACC1, c‐Met, and PDL1 were increased significantly after transfection with the pCDNA3.1(+) vector containing MACC1 coding sequences compared with the vector‐NC group (Figure 2A, *P* < .05). These results showed that the transfection was successful. Furthermore, a western blot assay showed that knockdown of MACC1 could inhibit c‐Met and PDL1 expression, and overexpression of MACC1 could induce c‐Met and PDL1 expression (Figure 2B,C, *P* < .05). These results indicated that there is actually some type of regulatory relationship among MACC1, c‐Met, and PDL1.

### 3.3 MACC1 regulates PDL1 expression through the c‐Met/AKT/mTOR pathway in gastric cancer cells

**Figure 3:**
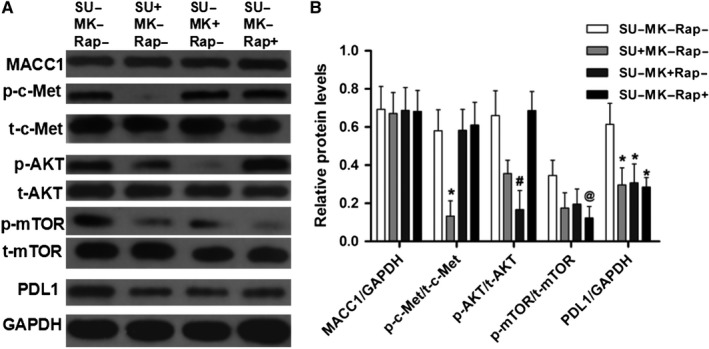
MACC1 regulates PDL1 expression via the c‐Met/AKT/mTOR pathway. (A) Western blot analysis of MACC1, p‐c‐Met, t‐c‐Met, p‐AKT, t‐AKT, p‐mTOR, t‐mTOR, and PDL1 expressions in MKN28 cells transfected with pCDNA3.1(+) vector containing MACC1 coding sequences. (B) The statistical results of western blot analysis. Data are the mean ± SD (n = 3), and each bar represents the mean of three independent experiments carried out in triplicate. *P < .05, compared with SU‐MK‐Rap‐ group. ^#^ *P* < .05, compared with SU‐MK‐Rap‐ group. ^@^ *P* < .05, compared with the SU‐MK‐Rap‐ group. SU: SU11274; MK: MK2206; Rap: rapamycin

c‐Met activation of a variety of tumor cell types plays an important role in regulating tumor progression,^11^ and c‐Met/AKT/mTOR pathway is involved in hepatocyte growth factor (HGF)‐promoted epithelial‐mesenchymal transition (EMT) and angiogenesis.^12^ To investigate the role of the c‐Met/AKT/mTOR pathway in c‐Met‐mediated PDL1 expression, we used SU11274, MK2206, and rapamycin to inhibit the c‐Met/AKT/mTOR pathway. The results are shown in Figure 3. Western blotting showed that three inhibitors significantly inhibited c‐Met, AKT, and mTOR phosphorylation, and all of the inhibitors reduced PDL1 expression compared with the no inhibitor (SU‐MK‐Rap‐) group (*P* < .05). These results indicate that MACC1 regulates PDL1 expression through the c‐Met/AKT/mTOR pathway in GC cells.

### 3.4 MACC1 regulates gastric cancer cell killing and cytokine concentrations through PDL1 in vitro

**Figure 4:**
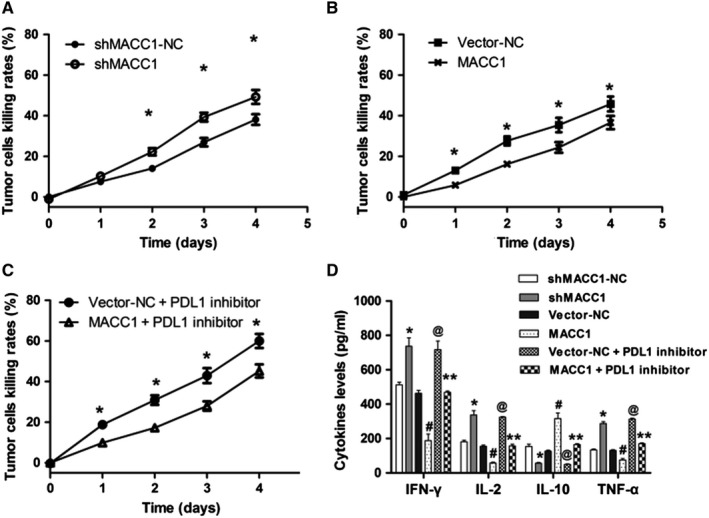
MACC1 regulates immune cell‐mediated gastric cancer killing and cytokine concentrations in vitro. (A) Silencing of MACC1 induces the immune cell‐mediated killing of gastric cancer cells. (B) Overexpression of MACC1 inhibits the immune cell‐mediated gastric cancer killing. (C) PDL1 inhibition is involved in the regulation of MACC1‐mediated immune killing. (D) ELISA analysis of IFN‐*γ*, IL‐2, IL‐10, and TNF‐*α* concentrations in cell supernatants. Data are the mean ± SD (n = 3), and each bar represents the mean of three independent experiments carried out in triplicate. *P < .05 and ^#^ *P* < .05, compared with shMACC1‐NC or vector‐NC group

When PDL1 levels were downregulated by shMACC1 transfection in MKN45 cells, the MTS assay indicated that the tumor cell killing rate of the shMACC1 group was significantly higher than that of the shMACC1‐NC group (Figure 4A, *P* < .05). When PDL1 levels were upregulated by MACC1 transfection in MKN28 cells, the MTS assay indicated that the tumor cell killing rate of the MACC1 group was significantly lower than that of the vector‐NC group (Figure 4B, *P* < .05). Furthermore, when MKN28 cells overexpressing MACC1 were treated with the PDL1 inhibitor, the tumor cell killing rates of the vector‐NC and MACC1 groups were significantly higher than those of the untreated vector‐NC and MACC1 groups (Figure 4C, *P* < .05).

When PDL1 levels were downregulated, an ELISA assay showed that the concentrations of IFN‐*γ*, IL‐2, and TNF‐*α* in the cell supernatant significantly increased in the shMACC1 group and markedly decreased in the MACC1 group compared with the shMACC1‐NC and vector‐NC groups, respectively (Figure 4D, both *P* < .05). In addition, concentrations of IL‐10 decreased in the shMACC1 group and markedly increased in the MACC1 group, compared with the shMACC1‐NC and vector‐NC groups, respectively (both *P* < .05). Furthermore, when MKN28 cells overexpressing MACC1 were treated with PDL1 inhibitor, the concentrations of IFN‐*γ*, IL‐2, and TNF‐*α* increased significantly both in vector‐NC + PDL1 inhibitor and MACC1 + PDL1 inhibitor groups, compared with the vector‐NC and MACC1 groups (*P* < .05). In addition, the concentrations of IL‐10 presented the opposite trend (*P* < .05).

These data show that MACC1 regulates immune cell‐mediated tumor killing and cytokine expression through PDL1 and that PDL1 is involved in immune cell‐mediated killing via MACC1 regulation.

### 3.5 Knockdown of MACC1 inhibits tumor growth, and overexpression of MACC1 induces tumor growth in vivo

**Figure 5:**
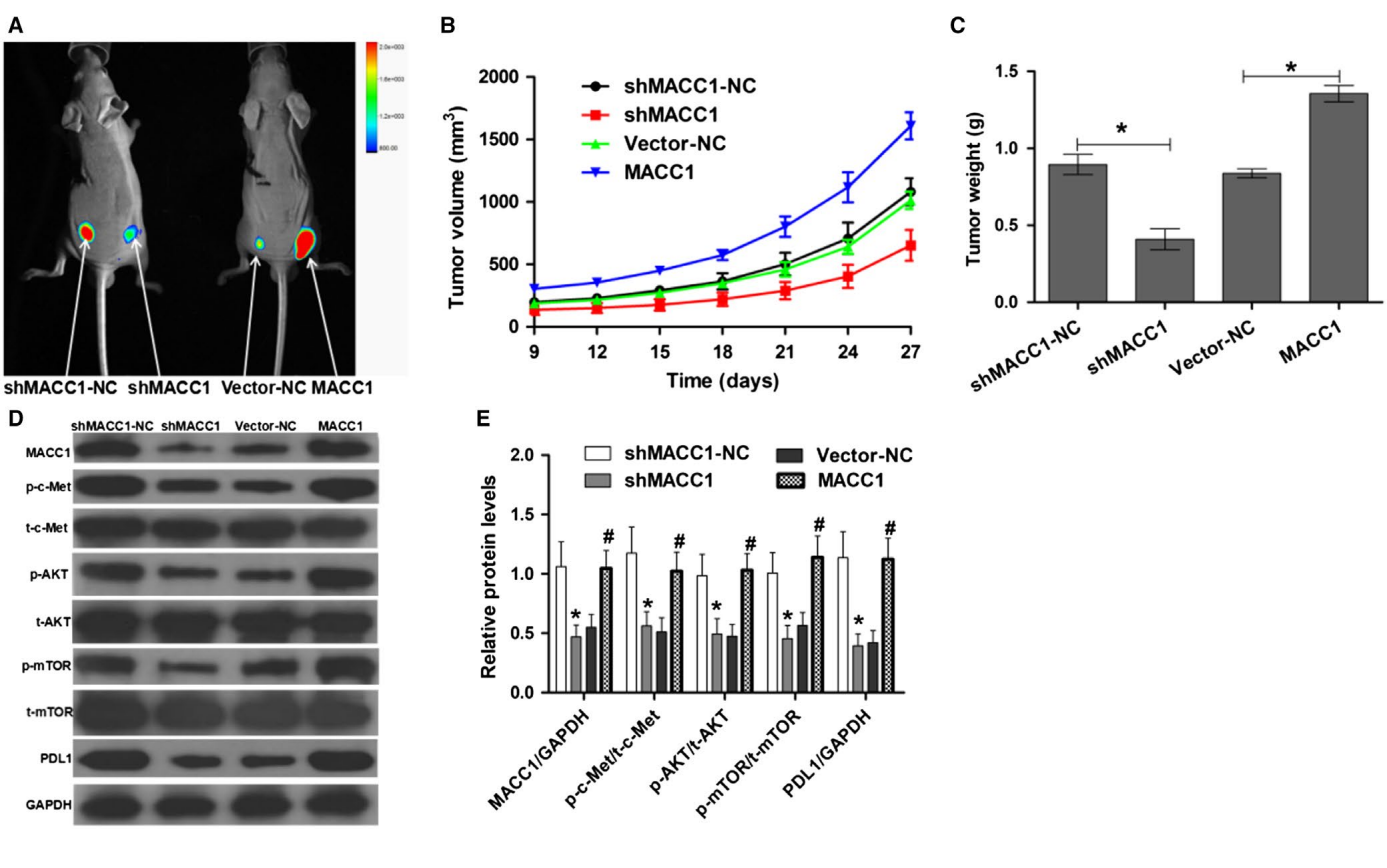
MACC1 regulates tumorigenesis in vivo. (A) Xenograft growth in nude mice. (B). Quantitative analysis of tumor volume. (C) Quantitative analysis of tumor weight. (D). Western blot analysis of MACC1, p‐c‐Met, p‐AKT, p‐mTOR, and PDL1 expression in mouse tumor samples. (E). The statistical results of western blot analysis. Data are the mean ± SD (n = 4), and each bar represents the mean of three independent experiments carried out in triplicate. *P < .05 and *^#^P* < .05, compared with shMACC1‐NC or vector‐NC group

To examine the roles of MACC1 in tumor progression, we constructed a BALB/C nude xenograft mouse model in which mice were transplanted with shMACC1‐NC and shMACC1 and vector‐NC and MACC1 stably transfected cell lines. After 4 weeks, tumors from the shMACC1 group were significantly smaller than those from mice transfected with shMACC1‐NC (Figure 5A), and silencing of MACC1 significantly reduced xenograft tumor volume (Figure 5B, *P* < .05) and tumor weight (Figure 5C, *P* < .05). In addition, tumors from the MACC1 group were significantly larger than those from the vector‐NC group (Figure 5A), and overexpression of MACC1 significantly increased xenograft tumor volume (Figure 5B, *P* < .05) and tumor weight (Figure 5C, *P* < .05). Furthermore, in vivo, silencing of MACC1 reduced the levels of phosphorylated c‐Met, AKT, and mTOR and PDL1 expression, and overexpression of MACC1 induced the phosphorylation of c‐Met, AKT, and mTOR and PDL1 expression (Figure 5D,E, *P* < .05).

### 3.6 MACC1 regulates the expression of cytokines in vivo

**Figure 6:**
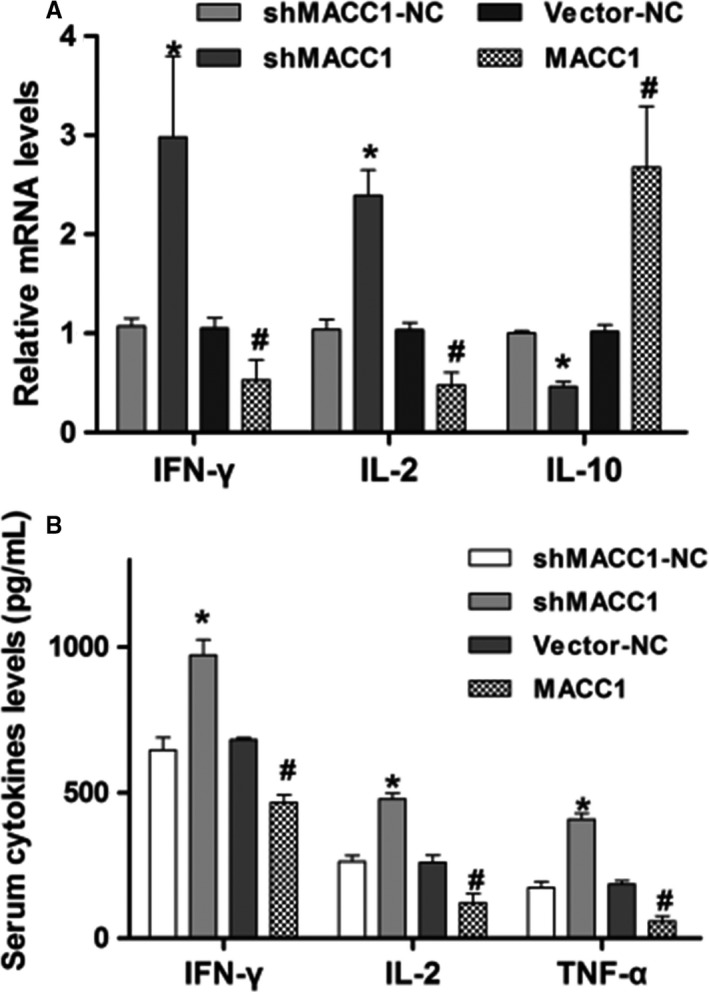
MACC1 regulates immune killing in vivo. (A) qRT‐PCR analysis of IFN‐*γ*, IL‐2, and IL‐10 expression in tumor tissue samples. (B) ELISA analysis of IFN‐*γ*, IL‐2, and TNF‐*α* concentrations in serum samples. Data are the mean ± SD (n = 4), and each bar represents the mean of three independent experiments carried out in triplicate. *P < .05 and *^#^P* < .05, compared with shMACC1‐NC or vector‐NC group

RT‐PCR showed that in comparison to those from the shMACC1‐NC and shMACC1 groups, the expression of IFN‐*γ* and IL‐2 was increased and the expression of IL‐10 was decreased in tumor samples from the shMACC1 group. Compared with those from the vector‐NC group, the expression of IFN‐*γ* and IL‐2 was decreased and the expression of IL‐10 was increased in the tumor tissue samples from the MACC1 group; this difference was significant (Figure 6A, *P* < .05). In addition, ELISA showed that the concentrations of IFN‐*γ*, IL‐2, and TNF‐*α* in mouse serum samples were significantly increased in the shMACC1 group and markedly decreased in the MACC1 group compared with the shMACC1‐NC and vector‐NC groups, respectively (Figure 6B, both *P* < .05). These data show that MACC1 regulates the expression of cytokines in vivo.

## 4 DISCUSSION

In this study, we found that MACC1, c‐Met, and PDL1 expressions were significantly higher in GC tumor tissues, and their expression levels were significantly associated with tumor grades. Furthermore, Pearson's correlation analysis indicated that there was a positive correlation between MACC1 and c‐Met expression levels, MACC1 and PDL1 expression levels, and c‐Met and PDL1 expression levels. For 98 GC and adjacent nontumorous tissues, Guo et al found that MACC1 expression was positively related to c‐Met expression.^13^ Cellular and molecular studies have indicated that c‐Met is a transcriptional target of MACC1 and that MACC1 acts as a key regulator of the HGF‐Met pathway.^14^ In renal cancer cells, Balan et al noted that c‐Met could regulate the expression of PDL1.^15^ The c‐Met/Akt/mTOR pathway is involved in cell migration and invasion in human lung cells.^16^ Other studies have also shown that c‐Met‐dependent PI3K/Akt/mTOR signaling pathways promote the development of solid tumors, such as glioblastoma and hepatocellular carcinoma.^17^ Based on these results and previous research, we hypothesized that MACC1 regulates PDL1 expression through the c‐Met/AKT/mTOR pathway in GC cells.

Using MACC1 overexpression and c‐Met/AKT/mTOR pathway inhibitors, we verified that MACC1 could regulate PDL1 expression through the c‐Met/AKT/mTOR pathway. SU11374, MK2206, and rapamycin are inhibitors of c‐Met, AKT, and mTOR phosphorylation, respectively. Their usage inhibited the activation of c‐Met/AKT/mTOR and led to a decrease in PDL1 expression. c‐Met is phosphorylated and subsequently activates motility, proliferation, invasion, and migration.^18^ Previous studies found that inhibition of c‐Met phosphorylation and its downstream cascade, such as suppression Akt phosphorylation and mammalian target of rapamycin (mTOR) phosphorylation, might provide new therapeutic strategies for various cancers.^16^, ^19^ Our study suggests that MACC1 regulates PDL1 expression via the c‐Met/AKT/mTOR pathway in GC cells.

The effects of PD‐1/PDL1 on tumor immunity have been studied in many reports. PD‐1/PDL1 interaction might promote tumor escape and play a negative role in tumor immunity,^20^, ^21^ probably because the PD‐1/PDL1 interaction can induce apoptosis of activated T cells.^22^ Iwai Y et al noted that PDL1 expression may be one of the mechanisms by which immunogenic tumors evade host immune responses, and blocking the interaction between PD‐1 and PD‐L may provide a promising tumor immunotherapy.^23^ PDL1 is commonly upregulated in response to proinflammatory cytokines, notably IFN‐*γ*, on many tumor cell types.^22^ In early clinical studies, blocking the PD‐1 pathway brought hopes for a tumor immunotherapy.^24^ Numerous studies have shown that anti‐inflammatory therapy is effective toward early cancer progression and malignant conversion, and inflammation offers an anticancer therapeutic opportunity.^25^ A variety of cytokines (IL‐2, IFN‐*γ*, IL‐6, TNF‐*α*, and so on) are involved in host‐mediated antitumor responses in vivo.^26^

In this study, the downregulation of PDL1 expression through MACC1 knockdown increased the GC‐killing ability of PBMCs, increased proinflammatory cytokines (IFN‐*γ*, IL‐2, and TNF‐*α*) expression, and reduced anti‐inflammatory cytokines (IL‐10) expression. Additionally, the upregulation of PDL1 expression decreased the GC cell‐killing ability of PBMCs, decreased proinflammatory cytokines (IFN‐*γ*, IL‐2, and TNF‐*α*) expression, and increased anti‐inflammatory cytokines (IL‐10) expression. In vivo, the downregulation of PDL1 expression inhibited tumorigenesis, and upregulation of PDL1 expression induced tumor progression. In addition, the upregulation of PDL1 expression decreased the expression of proinflammatory cytokines (IFN‐*γ*, IL‐2, and TNF‐*α*) in mouse serum and tumor tissue samples and increased the expression of anti‐inflammatory cytokines (IL‐10). Meanwhile, the downregulation of PDL1 expression increased IFN‐*γ*, IL‐2, and TNF‐*α* expressions in mouse serum and tumor tissue samples and decreased IL‐10 expression. These data show that PDL1 is involved in immune escape by GC cells and plays a negative role in tumor immunity.

In summary, we demonstrate that MACC1 regulates PDL1 expression and tumor immunity through the c‐Met/AKT/mTOR pathway in GC cells. MACC1 and the c‐Met/AKT/mTOR pathway may be therapeutic targets for GC immunotherapy.

## CONFLICT OF INTEREST

None declared.

## ETHICS STATEMENT

The study was approved by the Peking University Shenzhen Hospital. Written informed consent was obtained from the participants before this study. The use of gastric cancer tissue was approved by the above ethics committee.
